# Pre-transplant donor-reactive IL-21 producing T cells as a tool to identify an increased risk for acute rejection

**DOI:** 10.1038/s41598-021-91967-w

**Published:** 2021-06-14

**Authors:** Aleixandra Mendoza Rojas, Teun van Gelder, Ronella de Kuiper, Derek Reijerkerk, Marian C. Clahsen-van Groningen, Dennis A. Hesselink, Carla C. Baan, Nicole M. van Besouw

**Affiliations:** 1 Department of Internal Medicine- Nephrology & Transplantation, Erasmus MC Transplantation Institute, Erasmus MC, University Medical Center Rotterdam, Rotterdam, The Netherlands; 2 Department of Clinical Pharmacy & Toxicology, Leiden University Medical Center, Leiden, The Netherlands; 3 Department of Pathology, Erasmus MC Transplantation Institute, Erasmus MC, University Medical Center Rotterdam, Rotterdam, The Netherlands

**Keywords:** Kidney, Immunology, Transplant immunology, Translational research

## Abstract

Pre-transplant screening focuses on the detection of anti-HLA alloantibodies. Previous studies have shown that IFN-γ and IL-21 producing T cells are associated with the development of acute rejection (AR). The aim of this study, was to assess whether pre-transplant donor-reactive T cells and/or B cells are associated with increased rejection risk. Samples from 114 kidney transplant recipients (transplanted between 2010 and 2013) were obtained pre-transplantation. The number of donor-reactive IFN-γ and IL-21 producing cells was analyzed by ELISPOT assay. The presence of donor specific antibodies (DSA) was also determined before transplantation. Numbers of donor-reactive IFN-γ producing cells were similar in patients with or without AR whereas those of IL-21 producing cells were higher in patients with AR (p = 0.03). Significantly more patients with AR [6/30(20%)] had detectable DSA compared to patients without AR [5/84(5.9%), p = 0.03]. Multivariate logistic regression showed that donor age (OR 1.06), pre-transplant DSA (OR 5.61) and positive IL-21 ELISPOT assay (OR 2.77) were independent predictors of an increased risk for the development of AR. Aside from an advanced donor-age and pre-transplant DSA, also pre-transplant donor-reactive IL-21 producing cells are associated with the development of AR after transplantation.

## Introduction

The incidence of acute rejection (AR) has been reduced since the introduction of tacrolimus with mycophenolate mofetil combination therapy^1^. The multicenter European network EKiTE reported an AR rate of 16.2% within the first year after transplantation in kidney transplant recipients transplanted after the year 2005^2^. While this rate is much lower than previously reported rates (when as much as 50% of kidney transplantations was complicated by AR), AR still carries several risks for kidney transplant recipients^3^. Early AR is associated with histologically-proven graft inflammation up to two years after transplantation, an increased risk for the development of donor-specific anti-HLA class II antibodies (DSA)^4^ and overall higher rates of graft loss^5,6^. However, there is no consensus on the long-term impact of early AR, as several studies have not found higher graft failure rates in patients with early T cell-mediated rejection (TCMR)^7,8^.

Alloreactive T cells are considered to be key initiators and mediators in the process of allograft rejection. These alloreactive T cells are able to recognize human leukocyte antigen (HLA) peptides on the surface of donor antigen-presenting cells (APC) or processed and unprocessed donor allopeptides presented by recipient APC^9,10^. These pathways are recognized as the direct, indirect and semi-direct pathways of alloreactive T cell recognition, respectively. In addition, B cells are involved in several mechanisms that can contribute to the development of allograft rejection. These include antigen presentation, cytokine production and differentiation into alloantibody-producing plasma cells^11,12^. The latter mechanism requires T cells to co-localize with B cells in germinal centers within secondary lymphoid organs. Thereafter, specialized follicular T helper cells can assist antigen activated B cells to differentiate into antibody-producing plasma cells^13,14^. This germinal center reaction is also likely to occur in so called ectopic lymphoid structures (functionally similar structures to germinal centers), that can form within transplanted renal and cardiac allografts^15,16^.

Current pre-transplant screening mainly focuses on the detection of anti-HLA alloantibodies present in the serum of transplant candidates^17^. This strategy, however, does not account for the presence of donor-reactive memory T cells which may contribute to allograft rejection. A long-standing area of transplant research has focused on the development of in vitro methods that allow for the accurate detection of donor-specific T cell alloimmunity^18,19^. One such method was developed by Heeger et al., involving the use of an enzyme-linked immunosorbent spot (ELISPOT) assay in which donor-reactive memory IFN-γ producing T cells could be measured^20^. Since then, several research groups have found that the presence of pre-transplant donor-reactive IFN-γ producing cells is associated with the occurrence of early AR^21–25^. However, these findings could not be replicated in the Clinical Trials in Organ Transplantation-01 multicenter study, where no association between donor-specific pre-transplant IFN-γ ELISPOT and AR was found^26^. In 2019, Van Besouw et al*.* described an association between higher numbers of pre-transplant and post-transplant donor-specific interleukin (IL)-21 producing cells and AR in a case–control study^27^. IL-21 regulates the immune activity of different cells relevant in the setting of organ transplant rejection^28^. IL-21 enhances the cytotoxicity and production of the pro-inflammatory cytokine IFN-γ by natural killer (NK) cells and CD8+ T cells^29,30^, and it has also been shown to stimulate the expansion of Th17 cells^31,32^. Studies in heart transplant recipients have demonstrated that intragraft IL-21 mRNA levels were significantly increased in patients experiencing acute cellular rejection^33^. Additionally, IL-21 is crucial for T cell-dependent B cell differentiation into memory B cells and antibody-producing plasma cells. Due to the pleiotropic effects of IL-21, it is involved in both T cell-mediated rejection (TCMR) and antibody-mediated rejection (ABMR)^16,34–36^. The IL-21 ELISPOT assay is therefore, a promising assay for the detection and monitoring of donor-reactive T cells in transplant recipients. The aim of this study, was to assess whether donor-reactive IFN-γ and IL-21 producing T cells and B cell donor reactivity assessed pre-transplantation in kidney transplant recipients are associated with an increased rejection risk after transplantation.

## Materials and methods

### Study population

A cohort of 114 renal transplant recipients transplanted between 2010 and 2013 was sampled cross-sectionally within 24 h prior to transplantation. In order to be able to study more patients with a rejection, the study cohort was enriched with patients who experienced one or more rejection events after transplantation. All patients provided written informed consent and the study was approved by the Medical Ethical Committee of the Erasmus Medical Center in Rotterdam, the Netherlands (biobank protocol MEC-2010-022, MEC-2016-718). All transplantations were performed adhering to the Declaration of Istanbul and all experiments were performed in accordance with the relevant guidelines and regulations of our institution and in accordance with the ethical standards of the Declaration of Helsinki. No transplants from inmates were used. Kidney function was assessed by estimated glomerular filtration rate (eGFR, mL/min per 1.73 m2, calculated by the CKD-EPI equation), serum creatinine (umol/L) and urine protein-to-creatinine ratio (mg/mmol) until graft failure or until a follow-up period of 7 years after transplantation. At time of transplantation all patients had a negative complement-dependent cytotoxicity cross-match. Transplant recipients received induction therapy with basiliximab [Simulect; Novartis, Basel, Switzerland; 20 mg intravenously on days 0 and 4]. The post-operative immunosuppressive regimen after transplantation consisted of tacrolimus (Prograf; Astellas Pharma, Tokyo, Japan; aiming for pre-dose concentrations of 10–15 ng/mL in weeks 1–2, 8–12 ng/mL in weeks 3–4 and 5–10 ng/mL thereafter), MMF (Cellcept); Roche, Basel, Switzerland; starting dose of 1 g twice a day, aiming for pre-dose concentrations of 1.5–3.0 mg/L) and prednisolone. Prednisolone was tapered to 5 mg at month 3 and withdrawn at months 4–5. Only ‘for cause’ biopsies were performed in this patient cohort. Rejection was defined as biopsy-proven acute rejection (BPAR) within the first 6 postoperative months by a renal pathologist using 2 μm paraffin sections stained for HE, PAS, Jones and immunohistochemistry for C4d on 4 μm sections. After the completion of the study, all biopsies were reviewed again by a clinical pathologist (M.C.C.) in a blinded fashion and scored according to the Banff’15 classification^37^.

### Anti-HLA antibodies

Pre-transplant serum samples from recipients were screened for the presence of anti-HLA antibodies using the Lifecodes Lifescreen Deluxe (LMX) kit, according to the manufacturer’s manual (Immucor Transplant Diagnostics Inc. Stamford, CT, USA). Thereafter, anti-HLA class I (HLA-A, HLA-B, and/or HLA-C) or HLA class II (HLA-DR and/or HLA-DQ) antibodies were analyzed with a Luminex Single Antigen assay using LABscreen HLA class I and class II antigen beads (One Lambda, Canoga Park, GA, USA), as described in our previous study^13^. A cut-off mean fluorescence intensity value of 5000 was used to determine the presence of anti-HLA antibodies. The presence of donor-specific antibodies (DSA) was determined by comparing the measured HLA specificities with donor HLA typing.

### Peripheral blood mononuclear cells (PBMCs)

PBMCs were isolated from heparinized blood by density gradient centrifugation using Ficoll-Paque (GE Healthcare, Uppsala, Sweden). PBMCs were collected from the interphase, washed twice, and frozen in RPMI-1640 with glutamax (Life Technologies/Gibco BRL, Paisley, Scotland, United Kingdom) supplemented with 100 IU/mL penicillin (Lonza, Basel, Switzerland), 100 μg/mL streptomycin (Lonza), 15% heat-inactivated human serum, and 10% dimethyl sulfoxide (Merck KGaA, Darmstadt, Germany). The PBMCs were stored at − 140 °C until use.

### IFN-γ and IL-21 ELISPOT assay

As described in our previous study^27^ polyvinylidene fluoride (PVDF) plates (Millipore, Darmstadt, Germany) were coated with anti-human IFN-γ or IL-21 mAb (U-CyTech Biosciences, Utrecht, the Netherlands) overnight at 4 °C. Patient’s PBMCs were incubated with irradiated (40 Gy) PBMCs or spleen cells derived from the donor or irradiated third-party cells, which were completely HLA-mismatched with donor and recipient, in 200 μL culture medium [RPMI-1640 with glutamax (Life Technologies/Gibco) + 10% heat inactivated FBS (Biowest, Haarlem, The Netherlands) + penicillin + streptomycin (100 IU/mL penicillin, 100 IU/mL streptomycin; Lonza)]. Unstimulated patient’s PBMC served as negative control. Cells were incubated in the ELISPOT plate for 20 h (IFN-γ) or 44 h (IL-21) at 37 °C, 5% CO_2_, and 95% humidity to allow spot formation. Thereafter, the wells were washed with PBS, and biotinylated anti-human IFN-γ or IL-21 detection antibody (U-CyTech Biosciences) was added for a period of 2 h. After washing, the wells were incubated with streptavidin-HRP conjugate (U-CyTech Biosciences) for 1 h followed by AEC substrate (U-CyTech Biosciences) until distinct spots formed within 30 min. Color development was stopped by washing 3–5 times with water. When the ELISPOT plates were dry, spots were counted automatically by using a Bioreader 6000 ELISPOT-reader (Bio-Sys GmbH, Karben, Germany). In case of response in the unstimulated PBMCs, this response was subtracted from the stimulated response.

### Statistical analysis

Statistical analyses were performed using SPSS 21.0 (SPSS Inc, Chicago, IL, US) and figures were made using GraphPad Prism version 6.01 (GraphPad, Inc., La Jolla, CA). The Mann–Whitney *U*-test was used to analyze the number of IFN-γ and IL-12 producing cells between patients with and without rejection. Data are presented as median and interquartile range (IQR). Pearson’s chi-squared test was used to analyze the frequency of AR in patients with and without DSA. Receiver operating characteristic (ROC) curve analysis was used to calculate the cut-off value of number of donor-reactive IL-21 producing cells. Finally, multivariable binary logistic regression was performed to assess the odds ratio (OR) and 95% confidence interval (CI) for developing rejection. The regression was done using a stepwise backward selection method. A two-sided p-value ≤ 0.05 was considered statistically significant.

## Results

### Patient characteristics

In the study population of 114 patients, 30 (26.3%) patients experienced one or more rejections within the first six months after transplantation. First rejections were scored as 24 TCMR, 3 ABMR and 3 mixed TCMR and ABMR. Table 1 depicts the characteristics of the patients with and without rejection. Univariate analysis showed that donor age, historical PRA, presence of anti-HLA antibodies and DSA were significantly higher in patients who experienced rejection within the first 6 months after transplantation compared to non-rejectors.

**Table 1:** Patient characteristics.

KTX recipients	No rejection < 6 months	Rejection < 6 months	p-value
	84	30	
Age^a^, years, median (range)	51 (19–74)	52 (22–72)	ns
Male gender, *N* (%)	50 (59.5)	19 (63.3)	ns
**Donor**			
Living donor, *N* (%)	68 (81)	21 (70)	ns
Age^**a**^, years, median (range)	49.8 (21–73)	59.8 (24–86)	0.001
Cold ischemia time (H)	5.1 (1.50–26.97)	6.6 (1.07–2.50)	ns
Delayed graft function, *N* (%)	11 (13.1)	5 (16.5)	ns
**HLA mismatch, *N* (%)**			ns
0–2	32 (38.1)	8 (26.7)	
3–4	34 (40.5)	13 (43.3)	
5–6	18 (21.4)	9 (30)	
**Previous KTX, *N* (%)**			
Second/third	8 (9.5)/ 2 (2.4)	5 (16.7)/2 (6.7)	ns
**PRA %, mean (range)**			
Current	5.0 (0–80)	4.2 (0–35)	ns
Historical	13.5 (0–94)	26.3 (0–100)	0.03
**Time to first post-transplant biopsy, days, median (range)**	1077 (8–2116)	30 (6–169)	

^a^Age at transplantation.

*KTx* kidney transplantation.

### Donor-reactive IFN-γ and IL-21 producing T cells and rejection

**Figure 1:**
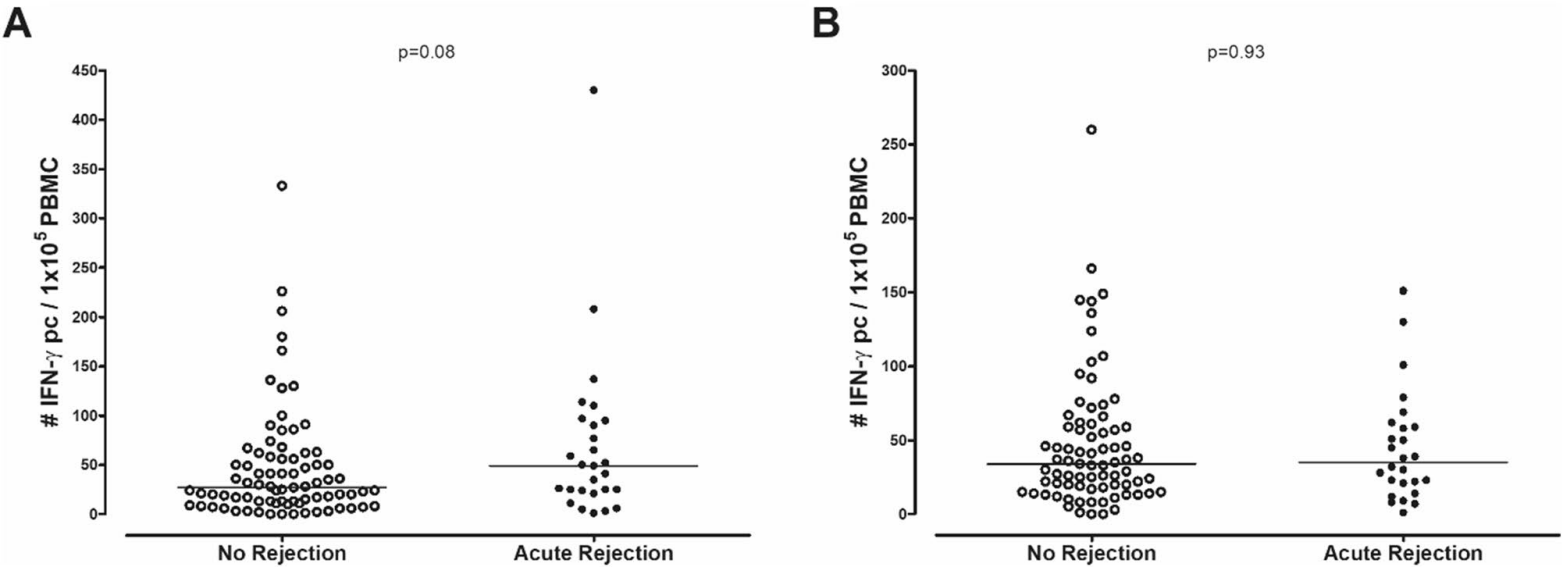
Number of donor-reactive IFN-γ producing cells (**A**) and third party-reactive IFN-γ producing cells (**B**) in n = 84 patients with no rejection and n = 30 patients with acute rejection.

**Figure 2:**
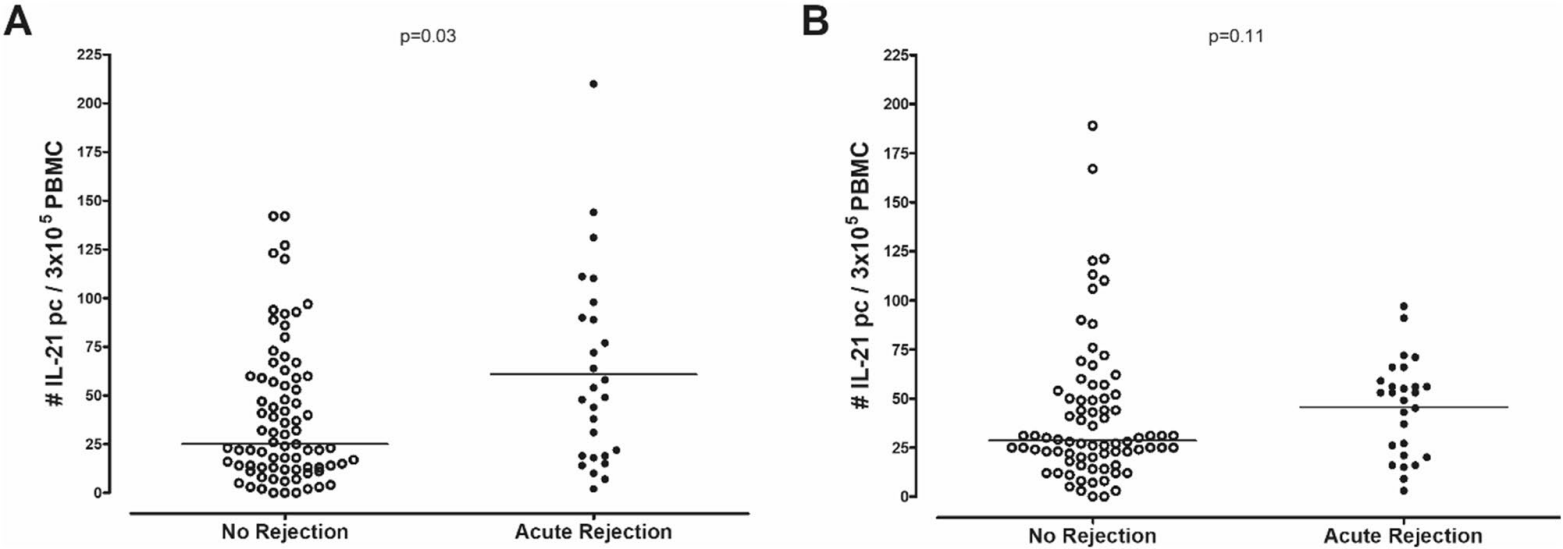
Number of donor-reactive IL-21 producing cells (**A**) and third party-reactive IL-21 producing cells (**B**) in n = 84 patients with no rejection and n = 30 patients with acute rejection.

**Figure 3:**
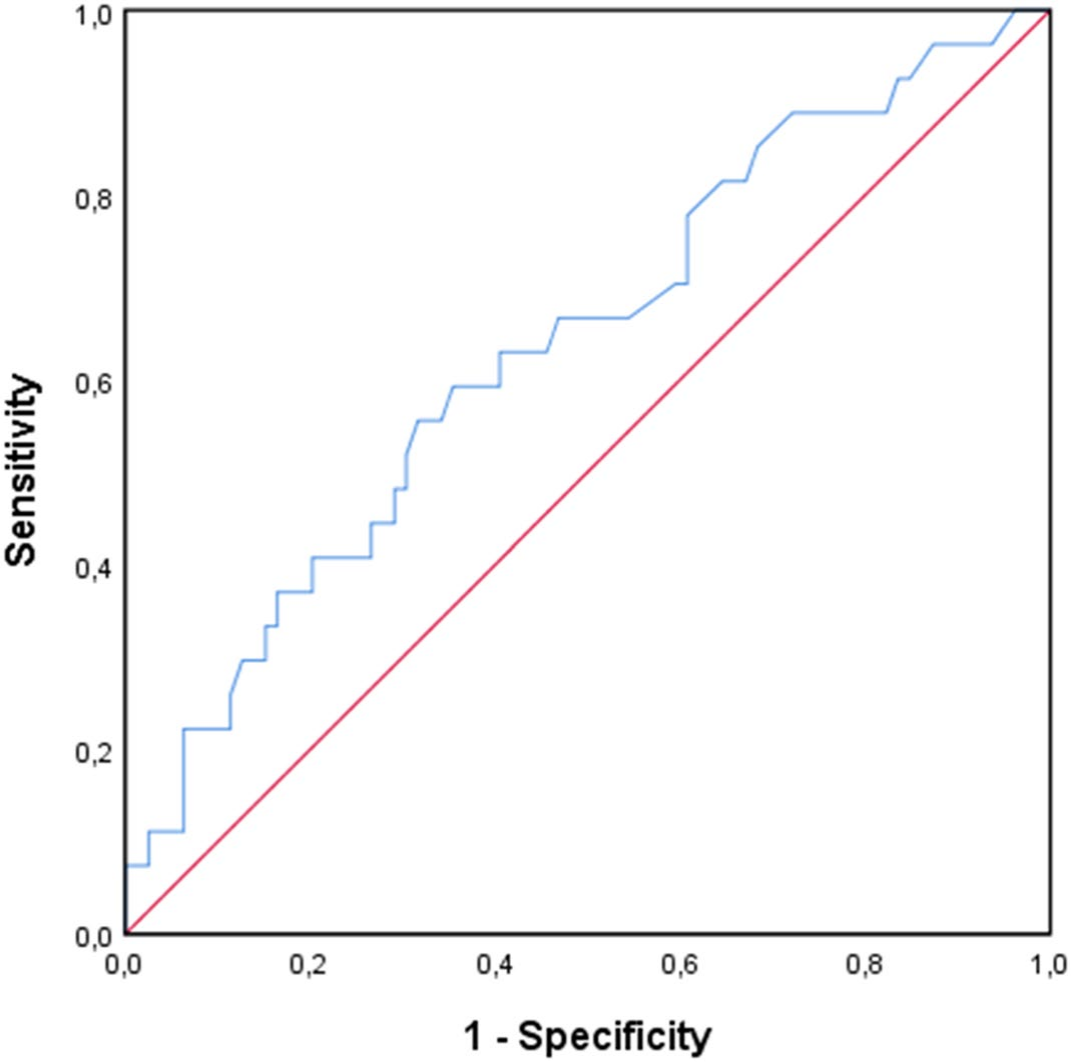
ROC curve of donor-reactive IL-21 ELISPOT assays to evaluate its performance in predicting acute rejection within 6 months after transplantation. A cut-off value of 37 donor-reactive IL-21 producing cells per 3 × 10^5^ PBMC resulted in an AUC of 0.64, this corresponds with a sensitivity of 63% and specificity of 60%.

The number of pre-transplant donor-reactive and third party-reactive IFN-γ producing cells was not significantly different between patients with and without AR [49/1 × 10^5^ PBMC (24–95) vs. 27/1 × 10^5^ PBMC (11–58); p = 0.08 and 35/1 × 10^5^ PBMC (19–60) vs. 34/1 × 10^5^ PBMC (18–60); p = 0.93, respectively, Fig. 1]. Patients who developed a rejection episode had significantly higher numbers of pre-transplant donor-reactive IL-21 producing cells compared to patients who did not develop rejection within 6 months after transplantation [49/3 × 10^5^ PBMC (19–90) vs. 25/3 × 10^5^ PBMC (13–59); p = 0.03, Fig. 2A]. There was no difference in third party-reactive IL-21 producing cells in patients who did or did not develop rejection [53/3 × 10^5^ PBMC (21–59) vs. 29/3 × 10^5^ PBMC (20–51); p = 0.11, Fig. 2B]. ROC analysis showed that a cut-off value of 37 donor-reactive IL-21 producing cells per 3 × 10^5^ PBMC resulted in an AUC of 0.64 and was able to discriminate patients with an early rejection with a sensitivity of 63% and specificity of 60% (Fig. 3). This cut-off also resulted in a positive predictive value (PPV) of 21.5% and negative predictive value (NPV) of 90.1%. These PPV and NPV were based on a rejection incidence of 15%. Because no association was found between IFN-γ and AR, no ROC curve was analyzed for this cytokine.

### B cell alloreactivity

At time of transplantation all patients had a negative complement-dependent cytotoxicity cross-match. Pre-transplant anti-HLA antibodies present in serum were measured for all patients. Out of 114 patients, 20 (17.5%) patients had detectable anti-HLA antibodies, of which 11 (9.6%) were donor-specific. A total of 4 patients had HLA class I DSA, 4 patients had HLA class II DSA and 3 patients had both HLA class I and II DSA. DQ DSA were the most prevalent, with 5/7 patients having measurable DQ DSA present prior to transplantation. The number of patients with a history of a previous kidney transplantation was significantly higher in patients who had detectable anti-HLA antibodies compared to patients with no anti-HLA antibodies, and the same was true for patients with and without detectable DSA (p < 0.001 and p < 0.001, respectively). Furthermore, significantly more patients with AR [6/30(20%)] had detectable DSA compared to patients without AR [5/84(5.9%), p = 0.03; OR 1.68, 95% CI 0.87–3.23].

### Donor-reactive IL-21 producing T cells and B cell alloreactivity

Patients with pre-transplant DSA did not have higher numbers of donor-reactive IFN-γ or donor-reactive IL-21 producing cells compared to patients without pre-transplant DSA. The association between a positive IL-21 ELISPOT assay (defined as 37 or more donor-reactive IL-21 producing cells per 3 × 10^5^ PBMC) with the presence of anti-HLA antibodies or DSA was analyzed. No significant differences in the presence of anti-HLA antibodies or DSA were observed between patients with and without a positive IL-21 assay.

### Clinical outcomes in patients with positive IL-21 ELISPOT assay

The relationship between a positive IL-21 assay and secondary clinical outcomes including eGFR, creatinine, proteinuria and time to graft failure were assessed. There was no significant difference in these graft function parameters from 1 to 7 years after transplantation in patients with a positive pre-transplant IL-21 ELISPOT assay compared to those with a negative IL-21 assay. A total of 48 patients experienced graft loss. The causes of graft failure were, death with a functioning graft (9/48), chronic rejection (23/48), acute rejection (7/48), vascular complications (3/48), recurrence of original disease (2/48), primary non-function (1/48) and other/unknown causes (3/48). The average time to graft failure in this selected population was 35.3 months. The average time to graft failure was significantly shorter in patients who had a positive pre-transplant IL-21 assay (27.9 months vs. 42.0 months, p = 0.02). Additionally, 13/31 (41.9%) patients with a positive pre-transplant IL-21 assay had a second and/or third rejection event during the follow up period of 7 years after transplantation. This rate of repeat rejections was significantly higher than in patients with a negative pre-transplant IL-21 assay (7/25, 28%; p = 0.04).

Furthermore, a multivariable binary logistic regression model was performed based on five predictive variables including donor age, historical PRA, pre-transplant anti-HLA antibodies, pre-transplant DSA and positive pre-transplant IL-21 assay. The results (Table 2) showed that donor age (OR 1.06, 95% CI 1.02–1.10), pre-transplant DSA (OR 5.61, 95% CI 1.21–25.88) and pre-transplant positive IL-21 assay (OR 2.77, 95% CI 1.04–7.36) were independent indicators of an increased risk for the development of rejection.

**Table 2:** Binary logistic regression.

	B	S.E	OR	95% CI for OR	p-value
Donor age^a^ , years, median (range)	0.06	0.02	1.06	1.02–1.10	0.004
Pre-transplant DSA	1.72	0.78	5.61	1.21–25.88	0.03
Pre-transplant IL-21 assay	1.02	0.5	2.77	1.04–7.36	0.04

## Discussion

The aim of this study was to investigate whether donor-reactive IFN-γ producing cells, donor-reactive IL-21 producing cells and B cell alloreactivity were associated with an increased rejection risk after kidney transplantation. We found that a higher donor age, the presence of pre-transplant DSA and a positive IL-21 assay were independent risk factors for the development of AR within 6 months after transplantation. Donor-reactive IL-21 producing cells have previously been found to be present in higher numbers in patients who develop AR^27^. Our data are in line with our earlier findings and this study confirms that a high number of donor-reactive IL-21 producing cells is a risk factors for AR. Both advanced donor age and the presence of pre-transplant DSA have been previously reported as being risk factors for a higher incidence of rejection and/or graft loss^38–41^. In particular, monitoring of pre-transplant as well as de novo DSA has become more widespread with an increasing number of studies focusing on the relationship between the presence of DSA and incidence of ABMR^42–45^. Although ABMR is often accompanied by the presence of DSA, this is not always the case, nor is the presence of DSA associated with adverse outcomes in all kidney transplant recipients^46–48^. While much remains unknown about the individual contributions of these risk factors, it is clear that donor-reactive immune memory can be difficult to detect and difficult to inhibit. Most research into the assessment of donor-reactive memory T cells, has been performed using the IFN-γ ELISPOT assay. In this study we found a trend towards a higher number of pre-transplant donor-reactive IFN-γ producing cells and AR. One possible explanation for the disparate findings is, that there are more patients with a humoral rejection component (20%) in our cohort compared to other studies which have primarily analyzed the association between donor-reactive IFN-γ producing cells in relation to acute cellular rejection^21–23,26^. As with other rejection biomarkers, implementation of the IFN-γ ELISPOT in clinical practice has been difficult due to its inability to predict clinical risk at the individual patient level. Similarly, the IL-21 ELISPOT assay may be limited in its capacity to accurately predict adverse clinical outcomes at an individual level, however, the high NPV of the assay may be of direct use in clinical practice. This may help to identify patients who are at reduced risk for the development of AR and may be ideal candidates for studies investigating the efficacy and safety of reduced intensity immunosuppressive protocols (for example in elderly patients). Following the data from van Besouw et al*.* published in 2019, this is the second study to find that a positive pre-transplant IL-21 ELISPOT assay is associated with AR. Due to the prominent role of IL-21 in both TCMR and ABMR, this assay may be well suited to predict several types of rejection. The IL-21 ELISPOT assay however, is not without its challenges. The assay has a relatively long incubation time of 44 h and requires the use of donor cells, as the predictive power was lost with third-party cells. Similar to other assays which require the use of donor cells, it is unlikely that results are available before a donor kidney from a deceased donor is transplanted. In its current form the assay is most useful for living donor transplants where PBMCs of the donor and the results of the IL-21 ELISPOT assay can be made available before the actual transplant is performed.

In addition to pre-transplant risk assessment, it would also be of great interest to assess the frequency of donor-reactive IL-21 producing cells during an active or chronic rejection event. The addition of the IL-21 ELISPOT assay to current pre-transplant screening may serve as a valuable addition to assess memory T cell reactivity, thereby providing a more complete view of the allogeneic immune response in transplant recipients.

## Data Availability

The raw data supporting the conclusions of this article will be made available by the authors, without undue reservation.
